# Psychological interventions for posttraumatic stress disorder involving primary care physicians: systematic review and Meta-analysis of randomized controlled trials

**DOI:** 10.1186/s12875-020-01244-4

**Published:** 2020-08-26

**Authors:** Rebekka Gehringer, Antje Freytag, Markus Krause, Peter Schlattmann, Konrad Schmidt, Sven Schulz, Sophie Jana Zezulka, Florian Wolf, Jonas Grininger, Mathias Berger, Horst Christian Vollmar, Jochen Gensichen

**Affiliations:** 1 Institute of General Practice and Family Medicine, Jena University Hospital, Friedrich-Schiller-University, Bachstr. 18, 07743 Jena, Germany; 2 Institute of Medical Statistics, Computer Sciences and Documentation, Jena University Hospital, Friedrich-Schiller-University, Jena, Germany; 3 Institute of General Practice/Family Medicine, University Hospital of LMU Munich, Munich, Germany; 4 Department of Psychiatry, Faculty of Medicine, University of Freiburg, Freiburg, Germany; 5 Department of Family Medicine, Ruhr-University of Bochum, Bochum, Germany

**Keywords:** PTSD, Primary care, Systematic review

## Abstract

**Background:**

Evidence-based psychological interventions for posttraumatic stress disorder (PTSD) are available in specialized settings, but adequate care in primary care is often lacking.

The aim of this systematic review was to determine the effectiveness of psychological interventions for PTSD involving primary care physicians (PCPs) and to characterize these interventions as well as their providers.

**Method:**

A systematic review and meta-analyses of randomized controlled trials (RCTs). Primary outcome were symptoms of PTSD.

**Results:**

Four RCTs with a total of 774 patients suffering from PTSD symptoms were included, all applying cognitive behavioural based interventions. Three studies with psychological interventions being conducted by case managers were pooled in a meta-analysis. Interventions were not effective in the short term (0–6 months; SMD, − 0.1; 95% CI, − 0.24-0.04; I^2^ = 0%). Only two studies contributed to the meta-analysis for long term (12–18 months) outcomes yielding a small effect (SMD, − 0.23; 95% CI, − 0.38- -0.08; I^2^ = 0%).

**Conclusions:**

Psychological interventions for PTSD in primary care settings may be effective in the long term but number and quality of included studies was limited so the results should be interpreted with caution.

## Background

Traumatic experiences can have long lasting effects on an individual’s mental and physical well-being including the developments of PTSD. There is an ongoing discussion about the characteristics of this disease [1], being reflected by the changes of its diagnostic criteria in the new diagnostic systems DSM-5 (Diagnostic and Statistical Manual of Mental Disorders 5th edition) and ICD-11 (International Statistical Classification of Diseases and Related Health Problems) [2, 3]. While ICD-11 requires three key symptom clusters of re-experiencing, avoidance and hyperarousal, DSM-5 added a fourth symptom cluster of persistent negative alterations in cognitions and mood.

PTSD lifetime prevalence is estimated between 1.3–8.8% in the World Mental Health Surveys [4]. The three most common causes of PTSD in the World Mental Health Surveys were rape, other sexual assault and unexpected death of a loved one [5].

Several evidence-based therapies for the treatment of PTSD are available to patients: cognitive- behavioural-based therapies (i.e. exposure therapies), eye movement desensitization and reprocessing (EMDR), and narrative exposure therapy (NET) [6]. Evidence suggests that trauma-focused therapies (i.e. exposure therapies, cognitive processing therapy, EMDR) may be more effective than non-trauma-focussed therapies [6, 7]. Pharmacological treatment using selective serotonin reuptake inhibitors may be helpful when trauma focused psychotherapies are not available or are refused by the patient [8]. Irrespective of the cause of trauma, most patients initially turn to general practitioners/primary care physicians (PCP – for reasons of consistency we refer to PCP in the further text) [9], with a median point prevalence of PTSD in primary care patients of 12.5% [10].

Despite the existence of effective interventions, there is a relevant time lag until patients receive specialist care. In the United States the median period of delay is 12 years between the onset of PTSD and first treatment contact (defined as talking about the disorder to any professional) [11]. Reasons for the delay in accessing treatment include the stigma associated with mental health care as well as cultural and institutional attitudes [12]. Additionally, there are structural impediments such as shortages of psychotherapists and inefficient allocation of patients to providers. To improve recognition and treatment of PTSD, several new approaches have been developed (e.g. internet based therapies, self-help, collaborative care) [13, 14].

National guidelines highlight that patients suffering from PTSD initially contact PCPs, who are in charge of diagnosing PTSD and organising care [15, 16]. There are several advantages in primary care settings which may facilitate the initiation and reduce barriers to psychological therapies: Treatment can be provided low-threshold and early starting compared to secondary care settings. Many patients have established a trustful relationship with their PCPs, thus an important first step within psychotherapeutic work has already been achieved [17]. PCP’s knowledge of their patients’ personal environment and resources can be an important resource during therapy [18, 19]. In addition, PCPs may help to close the large gap between supply and demand for the support of traumatized people [20].

Several other mental health disorders have effectively been treated in primary care, including depression and anxiety [21]. Important components for successful treatment include case management delivered by case managers (CM) with mental health training, scheduled supervision of CMs by mental health specialists, and the coordinated involvement of PCPs, CMs, and mental health specialists [22–24].

Two narrative reviews described the rationale for the management of PTSD in a primary care setting involving brief cognitive behavioural therapies (CBT); utilizing self-help and internet based approaches; as well as collaborative care [13, 14]. Based on a narrative review, Hoeft et al. suggest that collaborative care that offers psychotherapy is a promising approach [25]. The three reviews give an overview on diverse interventions investigated in a wide range of settings and with multiple designs. Given the heterogeneity of interventions, a quantitative analysis was not performed. A meta-analysis is needed to increase evidence on psychological treatment for PTSD in a primary care setting and to investigate specific intervention effects.

### Objectives

The aim of this review was to determine the effectiveness of psychological interventions for PTSD involving PCPs. The second aim was to characterize these interventions and their providers, and to describe the providers’ specific tasks, as well as their interaction.

## Methods

Objectives, inclusion criteria, and methods were pre-specified in a study protocol registered with Prospero (Registration number CRD42017060123).

### Eligibility criteria

Both cluster and individually RCTs were included applying psychological interventions to reduce PTSD symptoms. Participants aged 18 and older with a PTSD diagnosis (according to a valid diagnostic system, e.g. DSM-4 or 5, ICD-10) or with clinically relevant PTSD symptoms, determined by validated instruments, were considered.

Eligible interventions were: CBTs (cognitive therapy, cognitive processing therapy, cognitive restructuring therapy, coping skills training, exposure therapy including prolonged exposure and dialectical behavioural therapy), EMDR, NET and others such as writing therapy, hypnotherapy, interpersonal therapy, present centred therapy, eclectic psychotherapy and psychodynamic therapies [6, 26–30]. Application of case management only or other service delivery models such as collaborative care without the implementation of one of the above listed psychological interventions was excluded. To be eligible for inclusion, psychological therapies had to be delivered by PCPs, or by non-physician primary care providers, on condition that PCPs remained actively engaged in the intervention. Active engagement could mean treatment was linked to the monitoring, the clinical instructions, the supervision, or the advice of the PCP, or the therapist and the physician used a shared patient chart, or the PCP regularly received feedback on the therapy’s progress and made recommendations for further management. Control groups may have also received active interventions such as case management, training of PCPs and collaborative care approaches without the implementation of the above listed psychological interventions.

To be eligible the primary outcome had to measure PTSD symptoms using validated instruments.

We did not apply any restrictions concerning language, publication status, or year of publication.

### Search methods

The full electronic search strategy for Medline is published in additional file 1. To identify studies we searched electronic databases (Medline, Embase, PsycINFO, the Cochrane Central Register of Controlled trials and CINAHL) from their inception until November 2016. An update search was performed for the period from December 2016 to February 2019. Additionally, we screened several conference proceedings where content was available online (see additional file 1). We also searched the International Clinical Trials Registry Platform and reference lists from included studies and relevant reviews.

### Study selection and data extraction

Eligibility assessment was performed independently from RG and MK/SJZ. After screening abstract and title, 245 full texts were reviewed.

The data extraction form was developed on the basis of the EPOC data collection form and checklist, and the EQUATOR template for intervention description and replication (TIDieR) checklist [31, 32]. Due to the limited number of included studies the extraction form was piloted with one study. We received data from the main study and, if available, study design and protocols, as well as further publications reporting relevant outcomes. Two reviewers conducted outcome extraction independently (RG/JG). The primary outcome assessed was PTSD symptoms. Secondary outcomes recorded were comorbidities, quality of life, psychopharmacologic medication use, mental health care use, adverse events, patients` satisfaction, additional costs for intervention, treatment and medication adherence, and suicidality. The remaining data (i.e. quality criteria, components of the Chronic Care Model [33]) were extracted by RG and checked by MK. Additionally, we focused on the involvement of PCPs and extracted their profession, PTSD specific training, tasks performed, and their interaction with other providers. All disagreements occurring during study selection and data extraction were resolved through discussion. If no agreement could be reached, a team of authors made the decision (AF, RG, JG1, MK, SvS, KS, HCV)*.* When any information was missing the corresponding authors were contacted. 12 of the 22 contacted authors responded, but could not provide additional quantitative outcome data.

#### Risk of bias assessment

Risk of bias of included studies was assessed by two independent reviewers (RG/MK) using the Cochrane Risk of Bias Tool [34]. Resulting disagreements were resolved through discussion and with a third reviewer (AF).

The quality of evidence was assessed using GRADE [35].

### Statistical analysis

We estimated standardized mean differences with *Cohen’s d* due to different scales for PTSD symptoms among studies. Heterogeneity was quantified using I^2^-statistics and linear mixed models. Quantitative analyses were performed using R and SAS 9.4. Due to the absence of statistical heterogeneity, we used the fixed effects model for performing the meta-analysis. Because standard deviations for means of the primary outcome were neither reported nor could be calculated from the presented data in one study [36], and could not be provided by the authors, we used the reported standard deviations for baseline means also for the follow-up data. To additionally assess the effects of time, intervention, and time-intervention interaction on the primary outcome we applied a linear mixed model using a restricted maximum likelihood method on a Gaussian distribution. The model selection was based on the Bayesian information criterion (BIC). We chose the model with the lowest BIC. All calculations for regression analyses were performed using SAS (proc GLIMMIX) procedure.

Other than announced in the protocol, no further analyses (meta-regression, sensitivity analyses) were conducted because of the limited number of included studies. Publication bias was estimated considering the inclusion of small and negative effect studies. A funnel plot could not be developed because of the small number of studies included.

## Results

### Study selection and study characteristics

We identified 5996 records in electronic databases during our search (flowchart additional file 2). An additional 48 records were found through searches in reference lists, conference proceedings, and the International Clinical Trials Registry Platform. After removing duplicates we screened 4418 records. Two hundred fourty-five full-texts were assessed for eligibility. Finally, 4 RCTs (STEPS-UP: Stepped Enhancement of PTSD Services Using Primary Care [36], DESTRESS-PC: Delivery of Self Training and Education for Stressful Situations-Primary Care version [37], CALM: coordinated anxiety learning and management [38, 39], PE-PC: Prolonged Exposure for Primary Care [40]) were included in this review with 774 participants suffering from PTSD. All studies were multicentre trials.

We extracted data from 16 reports (including research protocols, study designs and analysis of secondary data).

Study characteristics (Table 1): STEPS-UP [36] was performed on active-duty military members with 80.9% male participants in US-military primary care clinics. Relevant PTSD symptoms were measured with the PCL-C (PTSD Checklist-Civilian version) for inclusion. Details of further inclusion and exclusion criteria of single studies are summarised in additional file 3. This was the only study reporting adverse effects and no case of adverse effects was noted [36]. The intervention was a stepped-care model based on CBT, using nurse-assisted, online or telephone self-management in Step 2, and the possibility of mental health specialists delivered psychotherapy in the last step (Table 2). Psychological therapy lasted 6–9 weeks. Applied strategies to improve treatment adherence were motivational interviewing (MI) and behavioural activation (BA). The control group received collaborative care as usual care, which had been implemented within the military health care system previously and consisted of prepared primary care practices, care management and enhanced mental health specialty. PCPs prescribed psychoactive medications in both groups.

**Table 1:** Study characteristics

Study	STEPS-UP	DESTRESS-PC	CALM	PE-PC
**Country**	US	US	US	US
**Setting**	18 Army primary care clinics	Dep. Defense and Veterans Affairs primary care clinics	17 primary care clinics	two military treatment facilities
**Population**	active duty US military members with PTSD and/or depression	recently deployed military service members/ veterans with PTSD	primary care patients with PD, GAD, SAD, PTSD or all 4	active duty military service members with (subthreshold) PTSD
**Male gender No. (%)**	539 (80.9%) male ^a^	65 (81.3%) male	290 (28.9%) male ^b^	50 (75%) male
**Age in years**	31.2^a^	36.5	43.5 ^b^	40
**Sample size IG/CG No.**	285/281^c^	43/37	33/28 ^d^	34/33
**Psychological intervention**	Online/ telephone delivered CBT-based therapies; other evidence based psychotherapies	CBT based nurse-assisted, online self-management tool	computerized CBT program tailored to the 4 specific anxiety disorders	brief Prolonged Exposure for Primary Care
**Involved providers**	CM, PCP, MHS	CM, PCP, MHS	CM, PCP, MHS	PCP, MHS
**PTSD diagnosis instrument**	PTSD Checklist- Civilian version (PCL-C)	Clinician administered PTSD scale (CAPS)	Mini International Neuropsychiatric Interview	PTSD Checklist-Stressor Specific version (PCL-S)
**PCL-C at baseline, IG/CG, mean (SD)**	58.5 (11.1)/57.7 (10.8) ^a^	55.16 (10.89)/ 58.56 (10.01)	57.15 (12.56) ^d^/ 56.90 (12.57)^d^	49,8 (12,8)/52,2 (14,1)e
**Psychiatric comorbidity outcome measures**	SCL-20 (depression), AUDIT (alcohol consumption), PHQ-15 (somatic disorder), BPI (pain)	PHQ-8 (depression), PHQ-15 (somatic disorder)	GADSS (GAD), PDSS-SR (PD), SPIN (SAD), PHQ-8 (depression), BSI (somatization and anxiety)	PHQ-9, BHM

*CM* care manager, *PCP* primary care physician, *MHS* mental health specialist, *CBT* cognitive behavioural therapy, *PD* panic disorder, *GAD* generalized anxiety disorder, *SAD* social anxiety disorder;

^a^ patients with PTSD and/or depression;

^b^ all anxiety disorders (PD, GAD, SAD, PTSD);

^c^ patients with PTSD;

^d^ patients who selected PTSD as their principal disorder

^e^ PTSD Checklist-Stressor Specific version (PCL-S)

**Table 2:** **Characteristics of the intervention**

Study		STEPS-UP				DESTRESS-PC	
IC/CG		IG			CG	IG	CG
Psychological intervention	Description of the intervention	*STEP 3:*	*STEP 2:*	*STEP 1:*	collaborative care without implementation of psychological therapies	CBT-based & stress inoculation training in a nurse-guided online patient self-management paradigm	low intensity CM and training of PCPs
		psychotherapy	CBT based self-management	care manage-ment (education, BA, MI)			
	who received the intervention	patients’ request, high risk patients, unresponsive to STEP 1 + 2, PCPs decision	patients who remain clinically symptomatic after 3–6 weeks	all patients	all patients	all patients	all patients
	who delivered the intervention	local MHS	CM	CM, PCP	CM, PCP, MHS	CM/computer program	CM/PCP
	method of delivery	in-person or via telephone	online or via telephone	via telephone, electronic messaging, in-person	via telephone,	online, via telephone, E-Mail, in-person	via telephone, E-Mail, in-person
	duration of the intervention	not reported	6–9 weeks	12 months	12 months	6-max. 10 weeks	not reported
	number of contacts	not reported	3–9	min. 12	min. 12	log in 3 times /week, number of CM-contacts not reported	3 telephone check-ins, risk assessment at weeks 2/4/6
	strategies applied to sustain/ improve treatment adherence	CM were trained in BA, problem solving and MI			Adherence was monitored	not reported	not reported
Pharmacological intervention	interventions for improved pharmacological treatment	see STEP 2	Expert training in pharmacologic treatment for PCPs	not reported	Stepped pharmacological treatment	no intervention	no intervention
	who prescribed medication	PCP			PCP	not reported	not reported
Study		CALM			PE-PC		
IC/CG		IG	CG		IG	CG	
Psychological intervention	Description of the intervention	computer-assisted CBT program	usual care by PCP, referral to MHS possible		brief Prolonged Exposure for Primary Care	minimal contact group	
	who received the intervention	patients could choose computer-assisted CBT medication, or both	all patients		all patients	all patients	
	who delivered the intervention	CM (ACS)	PCP, MHS		PCP, MHS	PCP, MHS	
	method of delivery	in-person (CBT), via telephone (follow-up)	in-person, via telephone		in-person	via telephone	
	duration of the intervention	10 to 12 weeks, symptomatic participants could receive up to 3 more steps (i.e., another 10–12 weeks) of treatment	not reported		30 min appointments delivered over 4–6 weeks	6 weeks	
	number of contacts	CBT: 6 to 8 weekly sessions	not reported		4	6	
	strategies applied to sustain or improve treatment adherence	ACS received didactics of MI	not reported		review by an independent clinician using adherence rating forms	not reported	
Pharmacological intervention	interventions for improved pharmacological treatment	single-session medication management training for PCPs using a simple algorithm, adherence monitoring by ACS for medication management	not reported		psychotropic medication should remain unchanged throughout the intervention	psychotropic medication should remain unchanged throughout the intervention	
	who prescribed medication	PCP	PCP		not reported	not reported	

*IG* intervention group, *CG* control group, *CM* care manager, *PCP* primary care physician, *MHS* mental health specialist, *ACS* anxiety clinical specialist, *CBT* cognitive behavioural therapy, *BA* behavioural activation, *MI* motivational interviewing

DESTRESS-PC [37] participants were recently deployed military service members and veterans. Most participants were male (81.3%). The study was set in Department of Defense and Veterans Affairs primary care clinics. The clinician administered PTSD scale (CAPS) was used for diagnosis. Similar to STEPS-UP, a CBT based, nurse-guided, online self-management paradigm constituted the intervention, which lasted 6–10 weeks. The control group received usual care which was optimized by training of PCPs in PTSD identification and treatment and basic care management including phone check-ins to monitor symptoms and feedback to providers.

The CALM trial [38, 39] was set in US-primary care clinics and included 28.9% male participants. Diagnosis instrument was the Mini International Neuropsychiatric Interview. The intervention consisted of a computer-assisted, face-to-face treatment for 10–12 weeks and could be extended up to 3 times. MI was used to increase treatment adherence. The control group received the usual PCP care with the option of mental health specialist referral.

PE-PC [40] was performed on active-duty military members with 75% male participants in two US military treatment facilities. Relevant PTSD symptoms were measured with the PCL-S (PTSD Checklist- Stressor Specific version) for inclusion. The intervention was based on a brief protocol for prolonged exposure developed for a primary care setting. Behavioural health consultants (BHCs) working in the primary care team were specially trained to deliver the intervention. Psychological therapy lasted 4–6 weeks. The control group was contacted weekly by the BHCs to monitor their status and was offered to receive PE-PC also after 6 weeks. The BHCs worked as a consultant to the PCP.

Involved providers (Table 3): PCPs always received feedback on the ongoing therapy and special training, except for PE-PC (no PCP training). They were responsible for the pharmacological treatment and were supervised by mental health specialists in two studies [36, 38, 39]. CMs (registered nurses, nurses with a bachelor or a master of science in nursing, psychiatric mental health nurse practitioners, social workers, counsellors and psychologists) had a central role in three studies [36–39]. They delivered or assisted the psychological therapies, coordinated care and communication between PCPs and mental health specialists, educated patients, delivered MI, BA and counselling, and monitored symptoms. Mental health specialists supervised CMs in three studies and pharmacological treatment in two studies [36, 38, 39]. In STEPS-UP they developed recommendations together with the CM and delivered psychotherapies for patients in STEP 3. Only in PE-PC the intervention was delivered by specially trained BHCs and no case management was applied. Due to the very different therapy concepts, this study was not included in the meta-analysis.

**Table 3:** **Involvement of treatment providers in the intervention**

Study	Treatment providers	Profession	Special training for intervention	Tasks and interaction with other providers	Supervision received
**STEPS-UP**	PCP	not reported	Expert training in the pharmacologic treatment of depression and PTSD	- provision of information related to treatment options - providing evidence based pharmacotherapy - selection of the next step for a patient’s treatment plan (with CM assistance)- implementation of central teams’ recommendations- receives feedback from CMs and the central team	%
	CM	RN; social workers counsellors	trained and coached weekly by telephone in BA, problem solving, and MI, training in the web-based intervention	- coordinating care between involved providers - improving patients activation and engagement in their care (education, MI, BA) - assistance of patients and PCPs in choosing treatment options - assistance with web-based or delivery of telephone CBT self-management	by MHS
	MHS	psychiatrists; psychologists; clinical social workers	trained in empirically validated psychotherapies for PTSD and depression	- delivery of empirically validated psychotherapy - review of patients’ medication - providing CM caseload reviews - training and supervision of CMs.	by psychotherapist
	central team	CM; psychiatrist; psychologist; administrative support	not reported	- coordination and supervision of the intervention - development of recommendations for PCPs - reformulation of CM engagement strategies - ensure appropriate medication	%
**DESTRESS-PC**	PCP	not reported	pre-study didactic training regarding management of and clinical tools for PTSD and associated conditions	- treatment of patients with feedback from CM	%
	CM/ DESTRESS nurse	RN; MSN; BSN; PMHNP-BC	not reported	- assistance with the web-based DESTRESS-PC interface - monitoring of compliance and symptom levels - reengagement of participants with ≥2 missed logons - providing updates of patients’ status to PCP and MHS	%
	MHS	not reported	not reported	- receives weekly updates from DESTRESS nurses	%
**CALM**	PCP	internists; family physicians	single-session medication management training using a simple algorithm	- remains the clinician of record - prescribed all medications	by psychiatrist
	ACS (CM)	social workers; RN; psychologists	- formal training applying the MINI- didactics for CBT program, MI, medication algorithm for anxiety	- providing eligibility assessment - delivery of computerized CBT program - monitoring of symptoms and adherence	by psychiatrist and psychologist
	MHS	psychiatrists; psychologists	not reported	- providing weekly supervision of ACS for diagnostic, CBT and medication management issues - providing medication consultation to PCPs.	%
**PE-PC**	PCP	not reported	not reported	member of the primary care team	%
	MHS	doctoral-level behavioral health providers (three civilian, one military psychologist)	full training workshop for PE; one to two training cases in the PE-PC intervention under close supervision	- follows up any missed appointments and attempts to reschedule - delivering PE-PC	by PI and independet clinician

*CM* care manager, *PCP* primary care physician, *MHS* mental health specialist, *ACS* anxiety clinical specialist, *RN* registered nurses, *MSN* Master of Science Nursing, *BSN* Bachelor of science in nursing, *PMHNP-BC* board certified Psychiatric Mental Health Nurse Practitioner, *BA* behavioural activation, *MI* motivational interviewing, *CBT* cognitive behavioural therapy, *PE* Prolonges Exposure, *PE-PC* Prolonged Exposure for Primary Care, *PI* primary investigator

#### Results of individual studies

**Fig. 1:**
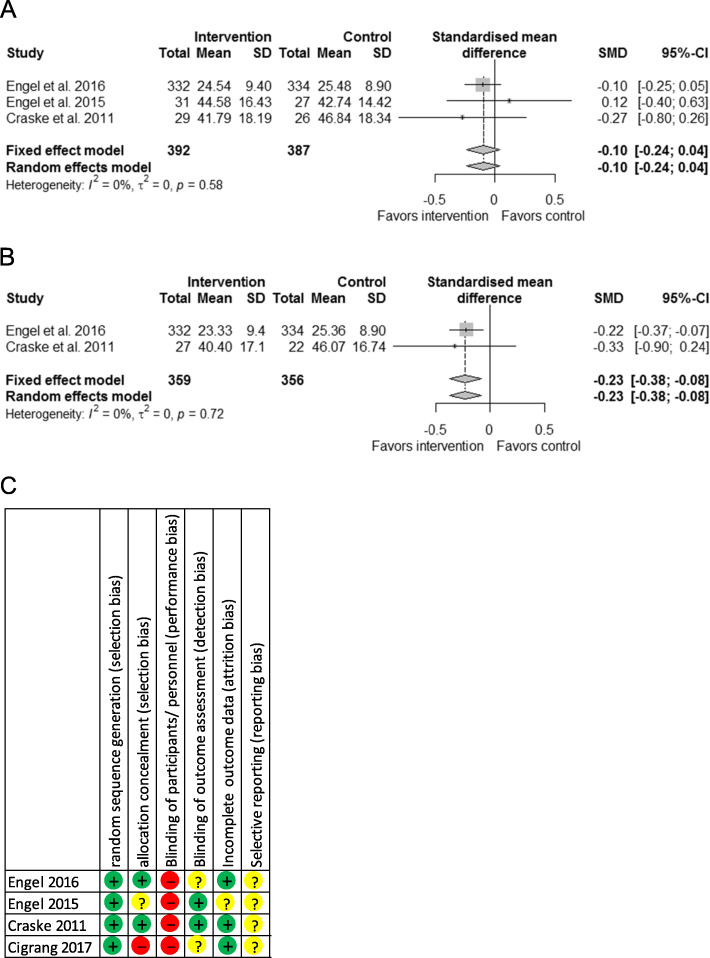
Meta-analysis of interventions for PTSD including psychological therapies in primary care vs. control; risk of bias summary. Small grey squares represent SMDs for PTSD symptom improvement of individual RCTs, large grey squares represent weights, horizontal lines show 95% CI, grey diamonds represent total SMDs of interventions and 95% CI. **a**: short-term effects (0–6 months); **b** long-term effects (12 months and longer); **c** risk of bias summary according to the Cochrane Collaboration Risk of Bias Tool

The individual results of three RCTs included in the meta-analysis in the short (0–6 months) and long term (≥ 12 months) are presented with the forest plot in Fig. 1.

#### Synthesis of results

In a meta-analysis we pooled the PTSD-symptom short-term (0–6 months) outcomes from three studies. According to the pooled analysis of the three studies, multifaceted interventions including psychological therapies and involving PCPs may make little or no difference to PTSD symptoms (SMD, − 0.1; 95% confidence interval, − 0.24-0.04; Fig. 1a). No statistical heterogeneity was detected (I^2^ = 0%; 95% CI, 0.0–80.8%). Only two studies reported on long-term outcomes (12–18 months). According to the meta-analysis of these two studies, psychological interventions in primary care may improve PTSD symptoms over time (SMD, − 0.23; 95% CI, − 0.38- -0.08; I^2^ = 0%; Fig. 1b). Because only two studies contributed to the pooled analyses for long-term effects, we also investigated time, intervention and time-intervention interaction effects with a generalized linear mixed model. Time (regression coefficient, −.28; SE, 0.05; *P* < .0001) and time-intervention interaction (regression coefficient, −.28; SE, 0.08; *P* = .0020) were significantly associated with means of PTSD symptom measures, with the treatment effect yielding a regression coefficient equal to .94; SE 0.52; *P* = .09.

We did not perform a meta-analysis for the treatment effects on comorbidities and quality of life due to the limited number of included studies. Further secondary outcomes were mostly not reported separately for the PTSD subgroup or not reported at all.

#### Risk of bias within studies

A summary of risk of bias judgments can be found in Fig. 1c. For a more detailed description, with support of judgments, see additional file 4. All studies had low risk of bias for random sequence generation. Allocation concealment was judged with low risk in two studies, with unclear risk and with high risk each in one study. Blinding of participants and personnel was not possible due to intervention study design. A lack of blinding may influence outcome, thus all studies scored high risk of bias. Blinding of outcome assessment was adequately reported in two studies, but risk was unclear in two studies. Two studies showed low risk of bias for incomplete outcome data. All studies did not report on all pre-specified secondary outcomes or no pre-specified outcomes were available at all and were judged with unclear risk for reporting bias.

#### Risk of bias across studies

Publication bias is difficult to judge with only four included studies. We performed a comprehensive search to reduce the risk of small studies not being detected. Two included studies are small in size and report on temporary effects only [37, 40].

#### Certainty assessment of evidence

Using GRADE we classified the quality of evidence as low because of a limited number of studies, the heterogeneous population of general primary care settings being not represented from the included study population and comparisons between heterogeneous intervention and control groups.

## Discussion

### Summary of evidence

We identified only four studies investigating psychological interventions for posttraumatic stress disorder (PTSD) involving primary care physicians (PCPs). According to our meta- and regression-analysis, multifaceted interventions including psychological therapies in primary care may make little or no difference in the short term, but may improve PTSD symptoms in the long term. These results should be interpreted with caution due to an overall low quality of evidence. All studies were conducted in the United States and three were in military settings. Case managers (CMs) had a central role in three interventions, which were pooled in a meta-analysis. They supported or conducted psychological therapies, coordinated communication between all treatment providers and provided patient activation through patient education, behavioural activation (BA), motivational interviewing (MI), and counselling. PCPs remained responsible for pharmacological treatment, received special training, and got regular feedback on ongoing therapy within the CM-based studies. One study did not involve CMs. The therapy (Prolonged Exposure) was delivered by specially trained behavioural health consultants (BHCs) who were part of the primary care team and worked as a consultant to the PCP.

There are two explanations for the small treatment effect on PTSD symptoms which was detected in the long term (after 12–18 months) but not directly after the intervention. One reason could be the lack of long term outcomes from DESTRESS-PC, the only study with a negative effect in the short term. Another explanation may be, that the long term effect after 12 months is a “learning effect”: While well-established psychological interventions are often short and intensive resulting in an immediate and strong effect, in primary care less intensive interventions are common with a lasting “learning effect”, which was also shown for collaborative care for depression [23, 41].

Though our aim was to determine the effectiveness of psychological therapies for PTSD in primary care, we only found studies investigating interventions which embedded psychological therapies into multifaceted service delivery models. Besides the applied CBT (cognitive behavioural therapy)-based psychological therapies also CM initialised patient activation (BA, MI, problem solving) and evidence based pharmacotherapy with adherence monitoring may have contributed to the detected positive treatment effect. Control groups were heterogeneous, too. The control group in STEPS-UP received collaborative care, which was previously implemented to improve PTSD primary care treatment within the military health care system. In DESTRESS-PC PCP training was also part of the optimized usual care. Interestingly, control groups of all three trials show a slight improvement of PTSD symptoms which may be caused by PCP training (DESTRESS-PC), CM alone (STEPS-UP) and mental health specialist referral (CALM). PCP training might be an important component because the ViStA trial (not included in our synthesis, comparing collaborative care for PTSD with minimally enhanced usual care) found that the intervention group (with CM and PCP training) and the control group (with PCP training) improved equally [42]. In depression care, training of PCPs resulted in no or only minimal improvement [24, 43], while feedback to PCPs about the ongoing therapy was associated with positive outcome measures [24].

The low effect size may partly be explained by the above discussed improvement of PTSD symptoms in the active control groups. In addition, the study population of the two largest trials (STEPS-UP and DESTRESS) consisted of military personnel, mainly men in their 20s, who are difficult to engage in mental health care [36, 37]. Finally the STEPS-UP population suffered from a variety of medical and psychiatric comorbidities reducing potential for improvement.

Our meta-analysis supports the explorative conclusion of a narrative systematic review suggesting collaborative care offering psychotherapy as a promising approach [25]. Looking at related diseases, short CBT interventions were effective for anxiety treatment in primary care settings, but results are heterogeneous for the effectiveness of psychotherapies in treating depression in primary care [21, 23, 24, 44]. Successful treatment of PTSD in primary care might be even more difficult than depression care: PTSD-symptom improvement was delayed compared to depressive symptoms [36] similar to previous studies showing that depressive patients with comorbid PTSD have a delayed positive response to collaborative care [45, 46]. An explanation for these findings could be that PTSD is often complicated by comorbidities, which need to be considered when designing new trials and interventions [47].

Another system of service delivery was investigated by the fourth included study [40]: the Primary Care Behavioral Health Model, with behavioural health providers integrated in primary care. In contrast to the other studies no CM and strategies to improve patient activation were applied; so it was not included within the meta-analysis. Despite the small size of the studied population (*n* = 67) the moderate to large effect sizes obtained up to six months in the follow up assessments encourage the idea that evidence based therapies for PTSD can effectively be transferred from secondary to primary care, especially for patients with mild to moderate symptom severity and enable an early starting therapy.

Because most mental health disorders are treated in general medical settings, primary care remains the first contact to establish effective therapies [48, 49]. In contrast to the central role of PCPs in many countries, in all four studies psychological therapies were provided with the assistance of a CM or by behavioural health providers, but were never delivered by the PCP in person. Only one pilot trial investigated the delivery of brief primary-care CBT delivered by a PCP, unfortunately without follow-up [50].

### Limitations

Our study has several limitations. The quality of evidence is low. There are only three studies contributing to the meta-analysis, with two studies including only a small number of patients with PTSD and one study with a short follow-up (4.5 months) limiting the generalizability of our findings. Performance bias was high in all studies. Due to study design it is difficult to blind participants and personnel for the intervention, thus introducing possible bias in subjective self-report measures. Assessing the certainty of evidence using GRADE [35] we detected serious indirectness because of applicability and indirect comparisons. The four included trials do not represent the heterogeneous population of general primary care settings. Three of four studies were conducted in military settings with mostly male patients who had experienced war-related trauma, which can be long-lasting, repetitive and may lead to complex PTSD. For this condition a sequenced or phase based multimodal therapy is recommended which can hardly be realised in primary care [51]. In non-specialised primary care settings the majority of PTSD patients may consist of female survivors of sexual assault [52].

Further indirectness arose from the multifaceted interventions and the heterogeneity in control groups as discussed above.

Reporting of results was often incomplete. In STEPS-UP a separate analysis of primary and secondary outcomes for patients with PTSD only (without depression) was missing. Study selection was difficult because descriptions of PCP involvement was insufficient and required us to contact several authors to request more detailed descriptions. Answers were not always available, so the risk remains that possibly eligible studies were not included.

Although statistical heterogeneity was not detected, all studies included in the meta-analysis show clinical diversity due to different settings (military vs. “general” primary care), different control groups (usual care vs. collaborative care vs. care management) and complex interventions including different components. Nevertheless, all studies applied CBT-based interventions with the help of a CM within a primary care setting and, therefore, it seemed appropriate to combine these studies.

The effectiveness of single intervention components could not be investigated with meta-regression due the lack of studies investigating only single components of the different service delivery models. The role of PCP involvement, especially, could not be assessed. The influence of interventions on comorbidities and quality of life could also not be calculated due to the limited number of included studies.

Our findings cannot be generalized to primary care settings in other countries because all studies were conducted in the United States and three studies were conducted in military contexts. In addition, the majority applied CM which is not always well established in other countries.

## Conclusion

To our knowledge this is the first systematic review and meta-analysis of primary care based psychological interventions for PTSD.

Four randomized controlled trails (RCTs) could be included which applied multifaceted interventions based on psychological therapies for PTSD involving PCPs. Psychological interventions for PTSD in primary care settings may be effective in the long term. Evidence supports the feasibility of primary care interventions for PTSD in general and the need for more studies examining psychological interventions for PTSD in primary care. The limited amount of research, an overall low quality of evidence and the rising number of different service delivery models in primary care require a differentiated analysis and hinder a universally valid recommendation for future treatment implications.

Some trials have only been published to date as study designs or pilot trials [53, 54], but may soon add relevant findings.

Future studies should investigate the contribution to effectiveness made by both intervention components and involved providers.

## Supplementary information

**Additional file 1.** Search strategy for Medline and conference proceedings searched.

**Additional file 2.** PRISMA Flow-Chart.

**Additional file 3.** Characteristics of included studies.

**Additional file 4.** Risk of bias in included studies.

## Data Availability

The datasets used and analysed during the current study are available from the corresponding author on reasonable request.

## Abbreviations

BA: Behavioural activation
BHC: Behavioural health consultant
BIC: Bayesian information criterion
CAPS: Clinician administered PTSD scale
CBT: Cognitive behavioural therapies
CCM: Chronic care model
CM: Case manager
DSM-5: Diagnostic and Statistical Manual of Mental Disorders 5th edition
EMDR: Eye movement desensitization and reprocessing
ICD: International Statistical Classification of Diseases and Related Health Problems
MI: Motivational interviewing
NET: Narrative exposure therapy
PCL-C: PTSD Checklist-Civilian version
PCL-S: PTSD Checklist-Stressor Specific version
PCP: Primary care physician
PTSD: Posttraumatic stress disorder
RCT: Randomized controlled trial
