# Supplementary material for: Psychological interventions for posttraumatic stress disorder involving primary care physicians: systematic review and Meta-analysis of randomized controlled trials
Source: BMC Fam Pract. 2020 Aug 26;21:176. doi: 10.1186/s12875-020-01244-4 (PMC7450546; doi:10.1186/s12875-020-01244-4)
Supplement: Supplementary file 1 — Additional file 1. Search strategy for Medline and conference proceedings searched. [file 12875_2020_1244_MOESM1_ESM.docx]

**Additional file 1: Search strategy for Medline and conference proceedings searched**

| #23 | Search (#22 OR #21 OR #16) |
| --- | --- |
| #22 | Search ((#2 OR #18) AND (#3 OR #17) AND #14 |
| #21 | Search (#20 AND #14) |
| #20 | Search (#17 AND #18 AND #19) |
| #19 | Search Adults[MeSH] |
| #18 | Search Stress disorders, traumatic[MeSH] |
| #17 | Search ((Primary Health Care[MeSH]) OR (General practice[MeSH])) |
| #16 | Search (#14 AND #15) |
| #15 | Search (#2 AND #3) |
| #14 | Search (#12 NOT #13) |
| #13 | Search (animals [mh] NOT humans [mh]) |
| #12 | Search (#4 OR #5 OR #6 OR #7 OR #8 OR #9 OR #10 OR #11) |
| #11 | Search groups [tiab] |
| #10 | Search trial [tiab] |
| #9 | Search randomly [tiab] |
| #8 | Search drug therapy [sh] |
| #7 | Search placebo [tiab] |
| #6 | Search randomized [tiab] |
| #5 | Search controlled clinical trial [pt] |
| #4 | Search randomized controlled trial [pt] |
| #3 | Search ((Primary Care[Title/Abstract]) OR (Family Physician*[Title/Abstract]) OR (General Practitioner*[Title/Abstract]) OR (GP[Title/Abstract]) OR (General Practice Physician*[Title/Abstract]) OR (Nurse Practitioner*[Title/Abstract]) OR (Primary Care Nurs*[Title/Abstract]) OR (Community Nurs*[Title/Abstract]) OR (Practice Nurs*[Title/Abstract]) OR (Health Care Assistant*[Title/Abstract]) OR (case manager*[Title/Abstract]) OR (General Practi*[Title/Abstract]) OR (Family Practi*[Title/Abstract]) OR (primary health care[Title/Abstract]) OR (outpatient*[Title/Abstract])) |
| #2 | Search ((((posttrauma*[Title/Abstract]) OR (post trauma*[Title/Abstract]) OR (PTSD[Title/Abstract]) OR (acute stress disorder[Title/Abstract]) OR (post-trauma*[Title/Abstract] ) OR (stress disorder*[Title/Abstract]) OR (combat disorder*[Title/Abstract] ) OR (war neuros*[Title/Abstract]))) OR (trauma*[Title/Abstract] AND (psycho*[Title/Abstract] OR stress*)[Title/Abstract])) OR (stress*[Title/Abstract] AND (extreme[Title/Abstract] OR disorder*)[Title/Abstract]) |

Conference proceedings searched: Conferences of the North American Primary Care Research Group, Society of Academic Primary Care, European General Practice Research Network, American Psychosomatic Society, Annual Meeting of International Society for Traumatic Stress Studies, *Deutsche Gesellschaft für Psychiatrie und Psychotherapie, Psychosomatik und Nervenheilkunde* (German Association for Psychiatry, Psychotherapy and Psychosomatics), annual meeting of *Deutschsprachige Gesellschaft für Psychotraumatologie* (German-speaking Society of Psychotraumatology), European Society for Traumatic Stress Studies
