# Supplementary material for: Psychological interventions for posttraumatic stress disorder involving primary care physicians: systematic review and Meta-analysis of randomized controlled trials
Source: BMC Fam Pract. 2020 Aug 26;21:176. doi: 10.1186/s12875-020-01244-4 (PMC7450546; doi:10.1186/s12875-020-01244-4)
Supplement: Supplementary file 2 — Additional file 2. PRISMA Flow-Chart. [file 12875_2020_1244_MOESM2_ESM.docx]

**Additional file 2: PRISMA Flow-Chart**

## Screening

## Eligibility

Records identified through database searching

Medline: n = 807

Embase: n = 690
Central: n = 2665

CINAHL: n = 478

PsycINFO: n = 1356

Additional records identified through other sources
(n = 48)

Records after duplicates removed
(n = 4418)

Records screened
(n = 4418)

Records excluded
(n = 4173)

Full-text articles assessed for eligibility
(n = 245)

Full-text articles excluded, with reasons
(n = 241)

No PTSD: 81

No psychological intervention in primary care: 132

Study design (no control, etc.): 22

Other reasons: 6

Studies included in qualitative synthesis
(n = 4)

Studies included in quantitative synthesis (meta-analysis)
(n = 3)

## Identification

## Included
