# Supplementary material for: Psychological interventions for posttraumatic stress disorder involving primary care physicians: systematic review and Meta-analysis of randomized controlled trials
Source: BMC Fam Pract. 2020 Aug 26;21:176. doi: 10.1186/s12875-020-01244-4 (PMC7450546; doi:10.1186/s12875-020-01244-4)
Supplement: Supplementary file 3 — Additional file 3. Characteristics of included studies. [file 12875_2020_1244_MOESM3_ESM.docx]

**Additional file 3: Characteristics of included studies**

| study |  | **Engel et al. 2016 (STEPS-UP)** |
| --- | --- | --- |
| methods |  | Six-site, two parallel arm randomized controlled trial of a stepped care approach compared to usual care with already implemented collaborative care |
| participants | inclusion, comorbidities | Patients recruited from 6 large army installations had to be on active duty. They had symptoms of at least moderate level on the PTSD Checklist, Civilian Version or depression (PHQ-9). Patients needed access to internet and email. Their mean age was 31 years, 81% were male and 48% were white, non-hispanic. Somatic symptoms were 13.8 (PHQ-15) and pain intensity was 5.7 (BPI). |
|  | exclusion | Current alcohol dependence, active suicidal ideation, major geographic relocation in the next 6 months, current duties in a participating clinic |
|  | setting | Primary care clinics at 1 of 6 military installations |
| intervention |  | Stepped care approach with STEP1: patient engagement, education, and preference development STEP2: Web-based self-management with CM assistance or telephone-based cognitive-behavioural therapy, evidence-based pharmacotherapy. STEP3: Local referral to specialty-based Mental Health Care |
| outcomes | patients | *symptoms*: PDS (posttraumatic diagnostic scale) *comorbidities*: somatic symptoms, alcohol abuse, health-related functioning, pain *therapy:* PTSD medication, CM contacts, individual therapy visits |
|  | providers | Qualitative interviews were conducted |
| study |  | **Engel et al. 2015 (DESTRESS-PC)** |
| methods |  | Two parallel arm randomized controlled trial of a nurse guided online self-management paradigm compared to low intensity care management |
| participants | inclusion, comorbidities | Patients had to be war veterans seeking care at a participating Department of Defense or Veterans Affairs primary care clinic, report war-related trauma during deployment, screen positive on a 4-item PTSD screener, and meet criteria for PTSD on the Clinician-Administered PTSD Scale (CAPS). Their mean age was 36 years, 81% were male and 55% were white, non-hispanic. Somatic symptoms were 12.4 (PHQ-15), comorbid anxiety disorder was assessed but not reported. |
|  | exclusion | Active engagement in trauma-focused mental health treatment; recent history of failed specialty mental health treatment for PTSD or an associated condition; acute psychosis, psychotic episode, or psychotic disorder; active substance dependence; active suicidal or homicidal ideation; currently taking antipsychotic or mood-stabilizing medication; unstable administration schedule or dosing of any antidepressant, anxiolytic, or sedative-hypnotic; acute or unstable physical illness |
|  | setting | Department of Defence and Veterans Affairs primary care clinics |
| intervention |  | CBT-based and stress inoculation training approaches in a nurse-guided online patient self-management paradigm |
| outcomes | patients | *symptoms:* PTSD Checklist, Civilian Version *comorbidities:* major depression, somatic symptoms, anxiety, health-related functioning *therapy:* treatment adherence |
|  | providers | - |
| study |  | **Roy-Burne et al. 2010 (CALM)** |
| methods |  | 4-site randomized controlled effectiveness trial of a computer-assisted CBT program (coordinated anxiety learning management) compared to usual care |
| participants | inclusion, comorbidities | Patients at participating clinics, 18 to 75 years, who met Diagnostic and Statistical Manual of Mental Disorders (Fourth Edition) criteria for 1 or more of panic disorder, generalized anxiety disorder, social anxiety disorder or PTSD (based on the Mini International Neuropsychiatric Interview) and scored at least 8 on the Overall Anxiety Severity and Impairment Scale (OASIS), Co-occurring major depression was permitted |
|  | exclusion | Persons unlikely to benefit from CALM (i.e., unstable medical conditions, marked cognitive impairment, active suicidal intent or plan, psychosis, bipolar I disorder, and substance abuse of dependence except for alcohol and marijuana abuse); Patients already receiving ongoing CBT or medication from a psychiatrist; persons who could not speak English or Spanish |
|  | setting | 17 primary care clinics |
| intervention |  | Computer-assisted CBT program (coordinated anxiety learning management) or medication or both |
| outcomes | patients | *symptoms*: PTSD Checklist, Civilian Version *comorbidities*: depression, anxiety disorders, Health-related functioning *therapy*: psychotropic medication type, dose, and adherence, number and consistency of CBT elements |
|  | providers | qualitative interviews were conducted |
| study |  | **Cigrang et al. 2017 (PE-PC)** |
| methods |  | Randomized controlled trial comparing brief, trauma-focused intervention developed for the primary care setting and a delayed treatment minimal contact control condition |
| participants | inclusion, comorbidities | Active duty military service members with: significant PTSD symptoms following military deployment, a PTSD Checklist-Stressor-specific Version score of 32 or higher, psychotropic medication stability for at least 4 weeks, and interest in receiving treatment for PTSD symptoms in primary care. Their mean age was 40 years, 75% were male. Depressive symptoms were 11,8 (PHQ-9) and their level of distress was 2,7 (BHM). |
|  | exclusion | Moderate or greater risk of suicide; severe traumatic brain injury; alcohol/substance use at a level that required immediate treatment; or current engagement in a trauma-focused behavioral treatment |
|  | setting | Primary care clinics of two military treatment facilities |
| intervention |  | Brief Prolonged Exposure for Primary Care |
| outcomes | patients | Symptoms: PTSD Checklist-Stressor Specific Version (PCL-S); PTSD Symptom Scale, Interview Version  (PSS-I)  Comorbidities: depression, general distress, life functioning |
|  | Providers |  |
