# Supplementary material for: Psychological interventions for posttraumatic stress disorder involving primary care physicians: systematic review and Meta-analysis of randomized controlled trials
Source: BMC Fam Pract. 2020 Aug 26;21:176. doi: 10.1186/s12875-020-01244-4 (PMC7450546; doi:10.1186/s12875-020-01244-4)
Supplement: Supplementary file 4 — Additional file 4. Risk of bias in included studies. [file 12875_2020_1244_MOESM4_ESM.docx]

**Additional file 4: Risk of bias in included studies**

| **Study** | **STEPS UP** |  |
| --- | --- | --- |
| Domain | Risk of bias | Support for judgment |
|  | Low/ High/Unclear |  |
| 1.     Random sequence generation (selection bias) | low | randomization by a computer automated system and stratification by site |
| 2.     Allocation concealment (selection bias) | low | automated emails were sent after permuted block randomization by a computer |
| 3.     Blinding of participants and personnel (performance bias) | high | participants and providers could not be blinded, outcome could be influenced by a lack of blinding |
| 4.     Blinding of outcome assessment (detection bias) | unclear | follow up assessments were completed using direct computer entry over the Internet, on-call behavioural health specialist team and the PI had access to the secure web portal |
| 5.     Incomplete outcome data (attrition bias) | low | ITT analysis, drop outs balanced among groups and reasons for drop outs mentioned |
| 6.     Selective outcome reporting? (reporting bias) | unclear | not all pre-specified secondary outcomes were reported |
| **Study** | **DESTRESS** |  |
| Domain | Risk of bias | Support for judgment |
|  | Low/ High/Unclear |  |
| 1.     Random sequence generation (selection bias) | low | stratification by healthcare site and gender, random permuted block scheme, central randomization |
| 2.     Allocation concealment (selection bias) | unclear | no information about how participants were informed about their allocation |
| 3.     Blinding of participants and personnel (performance bias) | high | participants and providers could not be blinded, outcome could be influenced by a lack of blinding |
| 4.     Blinding of outcome assessment (detection bias) | low | outcome raters were blinded |
| 5.     Incomplete outcome data (attrition bias) | unclear | no ITT for primary outcome, incomplete reasons given for drop outs |
| 6.     Selective outcome reporting? (reporting bias) | unclear | not all pre-specified secondary outcomes were reported |
| **Study** | **CALM** |  |
| Domain | Risk of bias | Support for judgment |
|  | Low/ High/Unclear |  |
| 1.     Random sequence generation (selection bias) | low | randomization by automated computer program, stratification by clinic and presence of comorbid major depression using a permuted block design |
| 2.     Allocation concealment (selection bias) | low | block size was masked to all clinical site study members. |
| 3.     Blinding of participants and personnel (performance bias) | high | participants and providers could not be blinded, outcome could be influenced by a lack of blinding |
| 4.     Blinding of outcome assessment (detection bias) | low | centralized telephone assessors blinded to treatment assignment. Incident report if a patient spontaneously mentioned their intervention status |
| 5.     Incomplete outcome data (attrition bias) | low | reasons for loss to follow up not provided and loss to follow up balanced among groups |
| 6.     Selective outcome reporting (reporting bias) | unclear | not all pre-specified secondary outcomes were reported |

| **Study** | **PE-PC** |  |
| --- | --- | --- |
| Domain | Risk of bias | Support for judgment |
|  | Low/ High/Unclear |  |
| 1.     Random sequence generation (selection bias) | low | block randomisation by web based application |
| 2.     Allocation concealment (selection bias) | high | participants were asked if they would like to receive PE-PC (Prolonged Exposure for Primary Care) by their behavioural health consultant, participants from the minimal contact control group received the intervention also after six weeks and the results were pooled |
| 3.     Blinding of participants and personnel (performance bias) | high | participants and providers could not be blinded, outcome could be influenced by a lack of blinding |
| 4.     Blinding of outcome assessment (detection bias) | unclear | not stated if the evaluator was blinded or not |
| 5.     Incomplete outcome data (attrition bias) | low | ITT analysis, drop out analysis for both groups and reasons for drop outs mentioned |
| 6.     Selective outcome reporting? (reporting bias) | unclear | no study protocol available |
